# Discovery of natural CdnP inhibitors through structure-based virtual screening and molecular dynamics simulations

**DOI:** 10.1128/spectrum.03258-24

**Published:** 2025-04-30

**Authors:** Xiaoxia Gu, Chaohu Xiong, Xinyu Wang, Hucheng Zhu, Weiguang Sun, Yan He, Jinwen Zhang

**Affiliations:** 1 Department of Pharmacy, Tongji Hospital, Tongji Medical College, Huazhong University of Science and Technology 731743, Wuhan, Hubei, China; 2 Hubei Key Laboratory of Natural Medicinal Chemistry and Resource Evaluation, School of Pharmacy, Tongji Medical College, Huazhong University of Science and Technology 593468, Wuhan, Hubei, China; Institute of Microbiology, Chinese Academy of Sciences, Beijing, China

**Keywords:** *Mycobacterium tuberculosis*, cyclic dinucleotide phosphodiesterase, host-directed therapy, immune evasion

## Abstract

Tuberculosis, caused by *Mycobacterium tuberculosis*, remains one of the most lethal infectious diseases both historically and in the post-coronavirus disease 2019 era. CdnP (Rv2837c) functions as a bifunctional oligoribonuclease and 3′(2′)-phosphoadenosine 5′-phosphate-phosphatase that impedes host immune responses by recognizing bacterial cyclic dinucleotides such as c-di-AMP, which serve as pathogen-associated molecular patterns. Despite its significance, strategies targeting CdnP remain limited. Through high-throughput virtual screening and enzymatic assays, we identified four natural product inhibitors: one coumarin derivative (macrosporusone A) and three flavonoid glucosides (ligustroflavone, rhoifolin, and neodiosmin). Surface plasmon resonance measurements confirmed direct binding of these compounds to CdnP with nanomolar to micromolar affinities. Molecular dynamics simulations elucidated a dual inhibitory mechanism wherein these compounds competitively occupy the product (AMP)-binding site while simultaneously constraining conformational plasticity of the substrate-binding domain. Evolutionary analysis demonstrated that these inhibitors exhibit broad-spectrum activity against bacterial CdnP orthologs while showing minimal inhibition of host-derived 2′,3′-cGAMP-specific phosphodiesterases, suggesting favorable selectivity. Notably, ligustroflavone exhibited superior inhibitory potency. In contrast, FDA-approved phosphodiesterase inhibitors showed poor activity against bacterial orthologs. These findings provide a foundation for developing novel host-directed therapeutics against tuberculosis that could potentially enhance stimulator of interferon genes (STING)-mediated immune responses without exerting selective pressure for antimicrobial resistance.

**IMPORTANCE:**

Tuberculosis (TB) remains a leading cause of mortality worldwide, with drug resistance posing a significant challenge to global control efforts. This study represents a major contribution to the field by identifying novel natural product inhibitors targeting CdnP (Rv2837c), a c-di-AMP-specific phosphodiesterase critical for *Mycobacterium tuberculosis* pathogenesis. The significance of this work lies in its innovative approach to TB therapy by perturbing bacterial nucleotide signaling pathways rather than directly inhibiting bacterial growth. By selectively targeting bacterial CdnP while avoiding host phosphodiesterases, these compounds—particularly ligustroflavone and other flavonoid glucosides—offer a promising foundation for developing host-directed therapeutics with potentially reduced selective pressure for antimicrobial resistance. Furthermore, the detailed structural insights and inhibitory mechanisms elucidated through molecular dynamics simulations provide valuable knowledge for rational drug design. This research bridges natural product discovery with computational biology to address the urgent need for novel TB treatments, especially against drug-resistant strains, presenting a significant advancement toward more effective therapeutic interventions for this persistent global health threat.

## INTRODUCTION

Tuberculosis (TB) claimed 1.25 million lives globally in 2023, re-emerging as a formidable public health threat following the decline of the coronavirus disease 2019 (COVID-19) pandemic. TB poses a particularly severe risk to immunocompromised individuals, notably those with HIV co-infection, a concern further amplified by the increasing prevalence of multidrug-resistant TB (MDR-TB). In 2023, approximately 10.8 million people worldwide contracted TB, necessitating an estimated annual expenditure of $22 billion in the USA alone for comprehensive TB prevention, diagnosis, treatment, and care programs (1). The eradication of the TB epidemic by 2030 constitutes a critical health objective within the framework of the United Nations Sustainable Development Goals.

*Mycobacterium tuberculosis*, the etiological agent of TB, produces the multifunctional enzyme CdnP (Rv2837c), which exhibits both oligoribonuclease and 3′(2′)-phosphoadenosine 5′-phosphate (pAp)-phosphatase activities. CdnP preferentially degrades RNA oligonucleotides, particularly dimers, and catalyzes the conversion of pAp to AMP (2). Significantly, CdnP acts upon a diverse range of substrates, including bacterial cyclic dinucleotides (CDNs) such as c-di-AMP and, with reduced efficiency, c-di-GMP, as well as host-derived CDNs like 2′,3′-c-GAMP. Both bacterial and host CDNs function as pathogen-associated molecular patterns, activating innate immune responses, particularly the stimulator of interferon genes (STING)-dependent type I interferon (IFN-α and IFN-β) signaling pathways (3). Furthermore, CDNs play crucial regulatory roles in numerous bacterial cellular processes, including biofilm formation, motility, virulence, central metabolism, osmotic regulation, DNA damage response, and sporulation (4–6).

Natural products have a distinguished history in antibiotic discovery and continue to offer a promising avenue for developing novel lead compounds and chemical entities for therapeutic applications (7–9). In this study, we aimed to identify novel anti-tuberculosis agents from natural sources. Through systematic screening of both in-house compound libraries and commercial resources, we identified four natural CdnP inhibitors: a coumarin derivative (macrosporusone A) and three flavonoid glucosides (ligustroflavone, neodiosmin, and rhoifolin). These compounds demonstrated inhibitory efficacy against CdnP that was comparable to or significantly exceeded that of FDA-approved phosphodiesterase (PDE) inhibitors. Furthermore, we elucidated the molecular mechanism of action of these inhibitors, establishing their potential as candidates for future development of anti-tuberculosis therapeutics.

## RESULTS

### Identification of natural CdnP inhibitors based on structure-based virtual screening

In pursuit of novel natural anti-tuberculosis agents, we systematically screened natural product compound libraries from both our laboratory collections and commercial sources. Our in-house library represents the culmination of years of dedicated research in natural product discovery (10–14). Commercial libraries were assembled by integrating collections from multiple suppliers and databases (including TargetMol, MedChemExpress, Alinda, Enamine, Thermo Scientific, Otava, Princeton BioMolecular Research, AnalytiCon, Vitas-M Laboratory, Specs, Asinex, and Mcule), with specific emphasis on natural product repositories. Given that CdnP functions as a dimer (5), our virtual screening strategy targeted the active site of one chain (chain A) within the dimeric CdnP model 5JJU, informed by previous findings that certain verified inhibitors are structural analogs of the substrate ApA (3). This approach yielded numerous promising candidates, from which we selected the top 100 compounds with the lowest docking scores for subsequent experimental validation.

**Fig 1:**
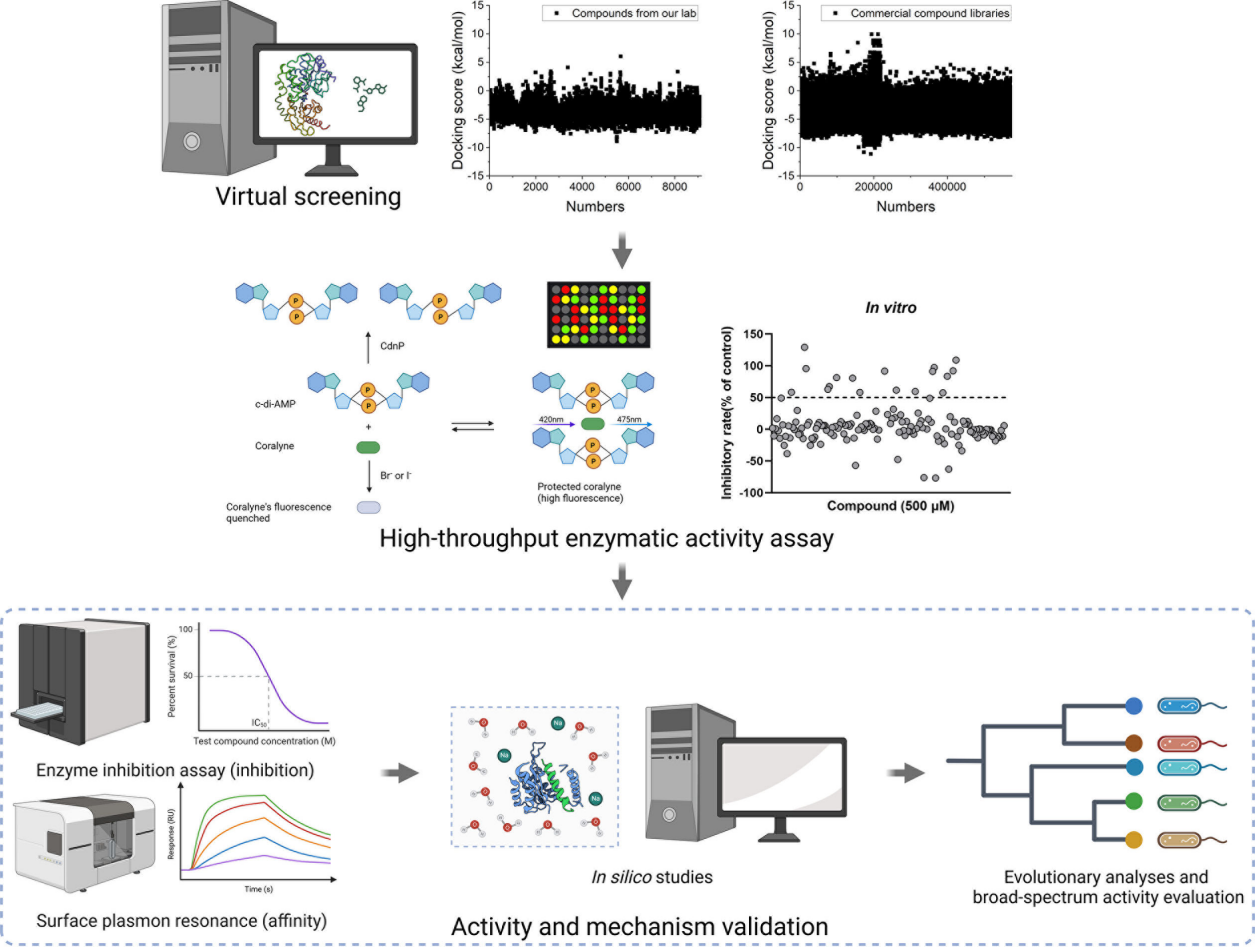
Schematic representation of the screening process for natural CdnP inhibitors. The screening protocol consisted of multiple sequential stages. Initially, virtual screening was conducted against natural compounds from both our in-house collection and commercial libraries. The secondary screening employed a high-throughput enzymatic activity assay, wherein c-di-AMP formed complexes with coralyne, protecting it from fluorescence quenching by halide ions. Potential inhibitors were identified by monitoring changes in fluorescence intensity. Subsequently, promising compounds underwent comprehensive validation through multiple complementary approaches: enzyme inhibition assays (quantifying inhibitory potency), surface plasmon resonance measurements (determining binding affinity), *in silico* studies, evolutionary analyses, and broad-spectrum activity evaluation.

Subsequently, we performed a secondary screening using a high-throughput enzymatic activity assay that leverages CdnP’s oligoribonuclease and pAp-phosphatase activities. The substrate c-di-AMP interacts with coralyne, protecting it from fluorescence quenching by halide ions while simultaneously enhancing coralyne’s fluorescence. In contrast, the hydrolyzed products (pApA or AMP) demonstrate no such protective effect. By monitoring these fluorescence changes, we identified potential CdnP inhibitors that exhibited significantly higher fluorescence intensity compared to control samples (Fig. 1).

This two-tiered screening approach confirmed four natural products as effective CdnP inhibitors: one coumarin derivative (macrosporusone A) and three flavonoid glucosides (ligustroflavone, neodiosmin, and rhoifolin) (Table 1).

**TABLE 1:** Chemical structures, sources, and inhibitory parameters of compounds exhibiting activity against CdnP

Compound	Chemical structure	Source	IC_50_^*a*^ (μM)	K_D_ (M)	Docking score (kcal/mol)
Macrosporusone A	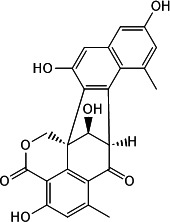	Talaromyces macrosporus (15)	16.82 ± 0.44	6.37e − 13	−5.682
Ligustroflavone	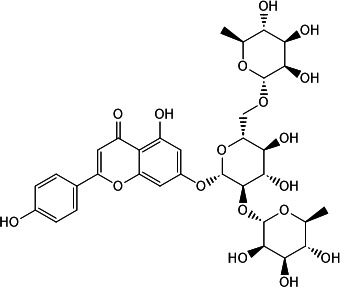	Ligustrum vulgare (common privet) (16–18)	39.54 ± 1.74	1.89e − 08	−8.046
Neodiosmin	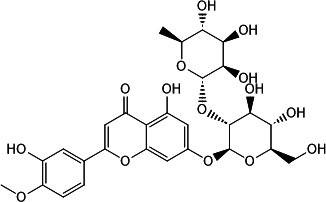	Citrus maxima (yu) (19)	5.50 ± 1.94	3.84e − 04	−7.152
Rhoifolin	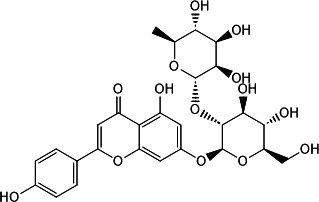	Multiple organisms (20–24)	20.32 ± 1.74	9.72e − 07	−8.990
Cilomilast	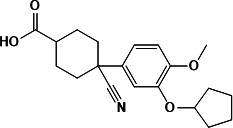	–^*b*^	> 1000	4.42e − 02	−5.378
Cilostazol	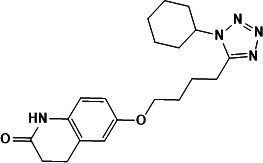	–	> 1000	4.18e − 06	−6.286

^*a*^ Half maximal inhibitory concentration.

^*b*^ Not applicable.

### Four natural products demonstrated potent CdnP inhibition

**Fig 2:**
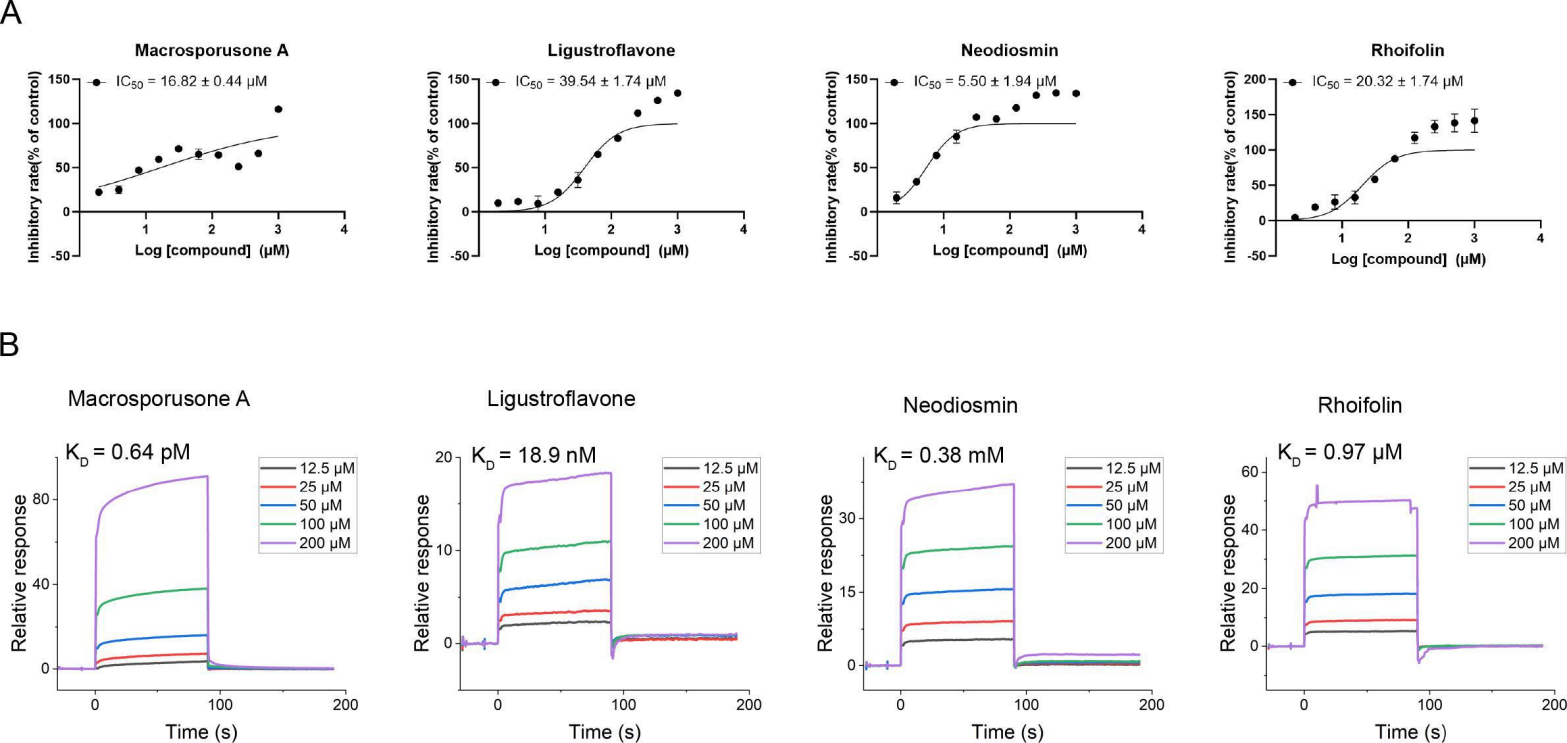
Validation of four natural products as effective CdnP inhibitors. (**A**) Dose-response curves illustrating the inhibitory activity (IC_50_) of four natural products against CdnP. (**B**) Surface plasmon resonance measurements demonstrating binding affinity between the four natural products and purified CdnP protein.

The inhibitory properties of the four identified natural products were validated by determining their IC_50_ values using an established high-throughput screening assay. All four compounds effectively inhibited CdnP, with neodiosmin exhibiting exceptionally potent activity (IC_50_ = 5.50 µM) (Fig. 2A, Table 1). While *M. tuberculosis* CdnP regulates both bacterial and host CDN levels, previous studies have shown that four FDA-approved PDE inhibitors—cilostazol (PDE3-I), cilomilast (PDE4-I), sildenafil (PDE5-I), and tadalafil (PDE5-I)—demonstrate mild inhibition of CdnP (3). In the absence of widely accepted reference compounds for CdnP inhibition assessment, these PDE inhibitors served as reference standards in our study. However, under our experimental conditions, these compounds showed no detectable inhibitory activity, even at concentrations as high as 1 mM (data not shown).

To further characterize the interaction between these compounds and CdnP, we employed surface plasmon resonance (SPR) to determine binding affinities. All four natural products demonstrated binding affinities ranging from micromolar to nanomolar concentrations, with macrosporusone A exhibiting exceptional affinity (K_D_ = 0.64 pM) (Fig. 2B, Table 1). These affinities significantly exceeded those of FDA-approved PDE inhibitors cilomilast and cilostazol (Fig. S1; Table S1). Notably, the remaining two PDE inhibitors showed no detectable binding to CdnP under identical experimental conditions.

### Molecular docking analysis of four natural products with CdnP

CdnP functions as a dimer, with the crystal structure 5JJU revealing a pApA molecule in chain A and two AMP molecules in chain B (5). We performed standard precision molecular docking of the four identified natural compounds and two PDE inhibitors (cilomilast and cilostazol) into both binding pockets of the 5JJU model. Our analysis demonstrated that all compounds exhibited preferential binding to the AMP-bound pocket rather than the pApA-bound site.

*In silico* docking simulations revealed that all four natural products displayed binding affinities equal to or exceeding those of the PDE inhibitors (Table 1), suggesting extensive interaction networks with CdnP. Notably, macrosporusone A established multiple stabilizing interactions within the active site, including two hydrogen bonds with Arg112 and Glu263, a π-π interaction with His132, and two coordination bonds with the catalytic Mn²^+^ ion.

The three flavonoid glucosides—ligustroflavone, neodiosmin, and rhoifolin—utilized their glucoside moieties to establish extensive polar interactions with the protein. Despite their structural similarities, these compounds adopted distinct binding orientations, resulting in diverse interaction profiles. Ligustroflavone, the largest compound examined, was predicted to form an extensive hydrogen-bonding network with multiple residues, including Arg112, Asp181, Glu263, Asp267, Lys282, Arg294, and His312. Additionally, it established an aromatic hydrogen bond with His131 and two π-π stacking interactions with Trp187.

Neodiosmin, which docked in a nearly perpendicular orientation relative to ligustroflavone, primarily interacted through hydrogen bonds with residues Ala46, Asp181, Leu213, Asp267, and Arg294. The modeling suggested a potential coordination bond between neodiosmin and one of the catalytic Mn²^+^ ions at the active site.

**Fig 3:**
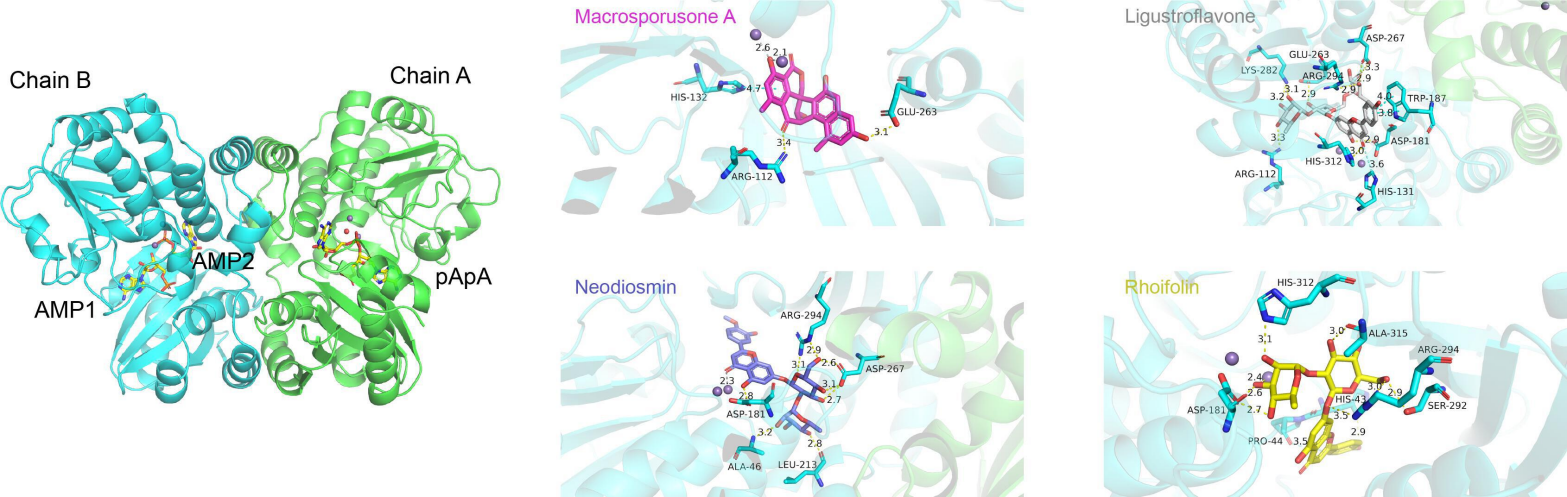
Molecular docking analysis of natural products with CdnP. The left panel depicts the dimeric structure of CdnP with its endogenous ligands (two AMP molecules in chain B and one pApA molecule in chain A). The right panel illustrates the predicted molecular interactions between the four identified natural product inhibitors and the CdnP active site.

Despite its structural similarity to neodiosmin, rhoifolin exhibited a markedly different binding conformation. It interacted with CdnP predominantly through hydrogen bonds with residues His43, Asp181, Ser292, Arg294, His312, and Ala315. Furthermore, computational analysis indicated an aromatic hydrogen bond between rhoifolin and Pro44 (Fig. 3).

### Molecular dynamics (MD) simulations of interactions between natural products and CdnP

To elucidate the mechanistic basis of inhibition by the four natural CdnP inhibitors, we performed MD simulations examining interactions between these compounds and CdnP, as well as the apo and endogenous ligand-bound states of the enzyme. Initial coordinates for the MD simulations were derived from molecular docking results. The various CdnP states were designated as follows: apo (ligand-free state), com_lig (complex with endogenous ligands), com_macro (complex with macrosporusone A), com_ligus (complex with ligustroflavone), com_neo (complex with neodiosmin), and com_rho (complex with rhoifolin).

**Fig 4:**
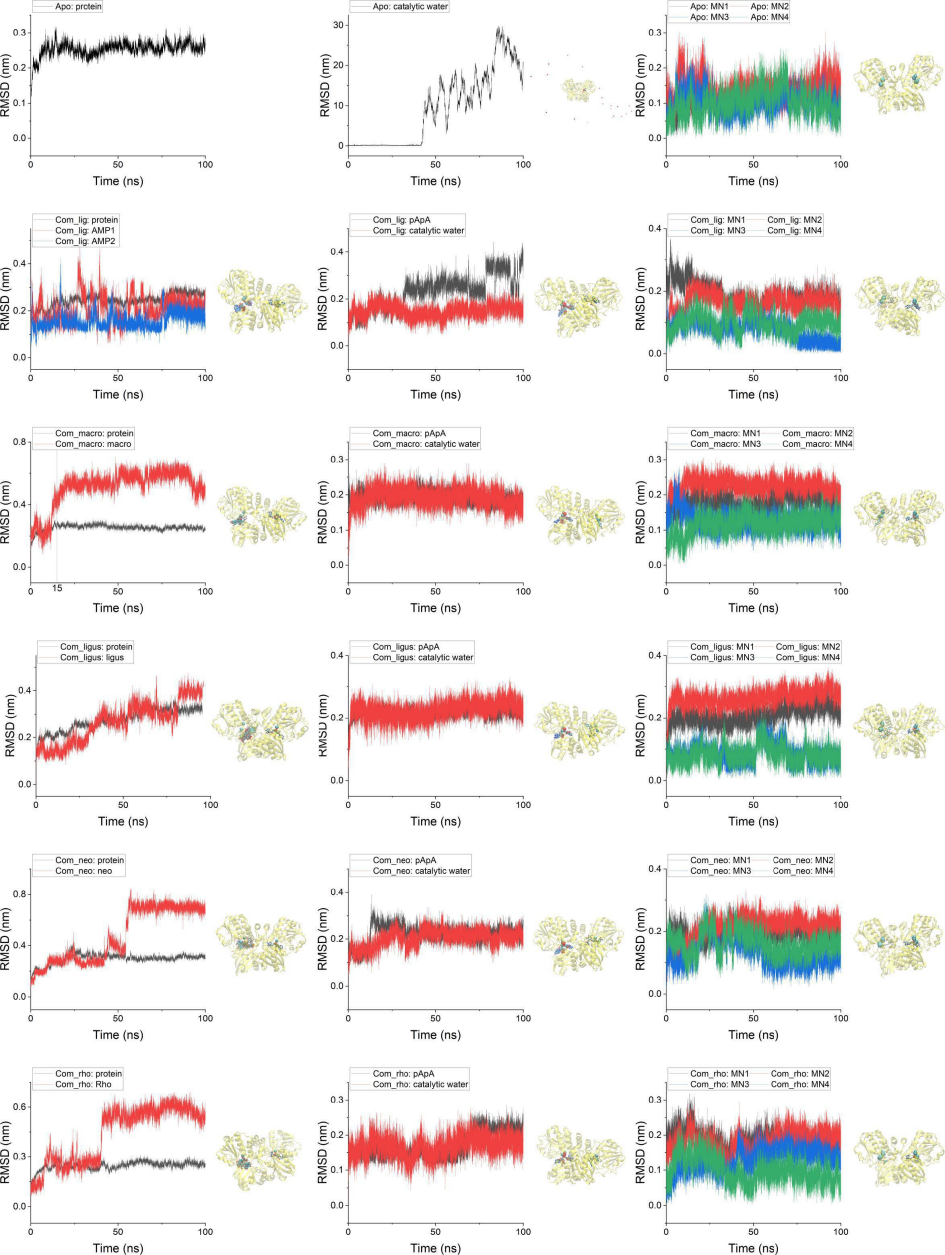
Conformational dynamics of CdnP in apo state and ligand-bound complexes. RMSD analysis of CdnP in its apo state and five distinct ligand-bound complexes, with corresponding molecular trajectories shown in the right panel. In the visualization, CdnP is represented as a yellow cartoon structure. Endogenous ligands and inhibitory compounds are depicted as licorice models colored by element, while the catalytic water molecule and four Mn²^+^ ions are represented by red and blue-green spheres, respectively. Trajectories of all components except CdnP are displayed following alignment to the protein structure.

Throughout the 100 ns simulation period, all ligands (both endogenous ligands and inhibitory compounds) maintained stable binding within the CdnP active site. Despite minor fluctuations observed in root mean square deviation (RMSD) calculations following CdnP alignment, the ligands remained confined to their respective binding pockets, as evidenced by ligand trajectory analysis and measurement of distances between the ligands and Asp181 in chain B of the active site (Fig. 4; Fig. S3A). Notably, the pApA molecule and its associated catalytic water molecule maintained stable positioning within the active pocket of chain A, contrasting with the apo state wherein the catalytic water dissociated into solution—a difference potentially attributable to increased backbone flexibility in the apo state (discussed below). CdnP coordinates two Mn²^+^ ions within each monomer. Upon ligand binding, these four Mn²^+^ ions exhibited asymmetric behavior, with ions proximal to chain A (pApA-bound) demonstrating greater conformational fluctuations than those associated with chain B (AMP or compound-bound). This asymmetric behavior likely reflects inherent structural differences between the two monomers, particularly within their respective binding pockets, during catalytic processes. Nevertheless, all Mn²^+^ ions maintained their positions within the active pockets without significant displacement from their original coordinates (Fig. 4, right column).

**Fig 5:**
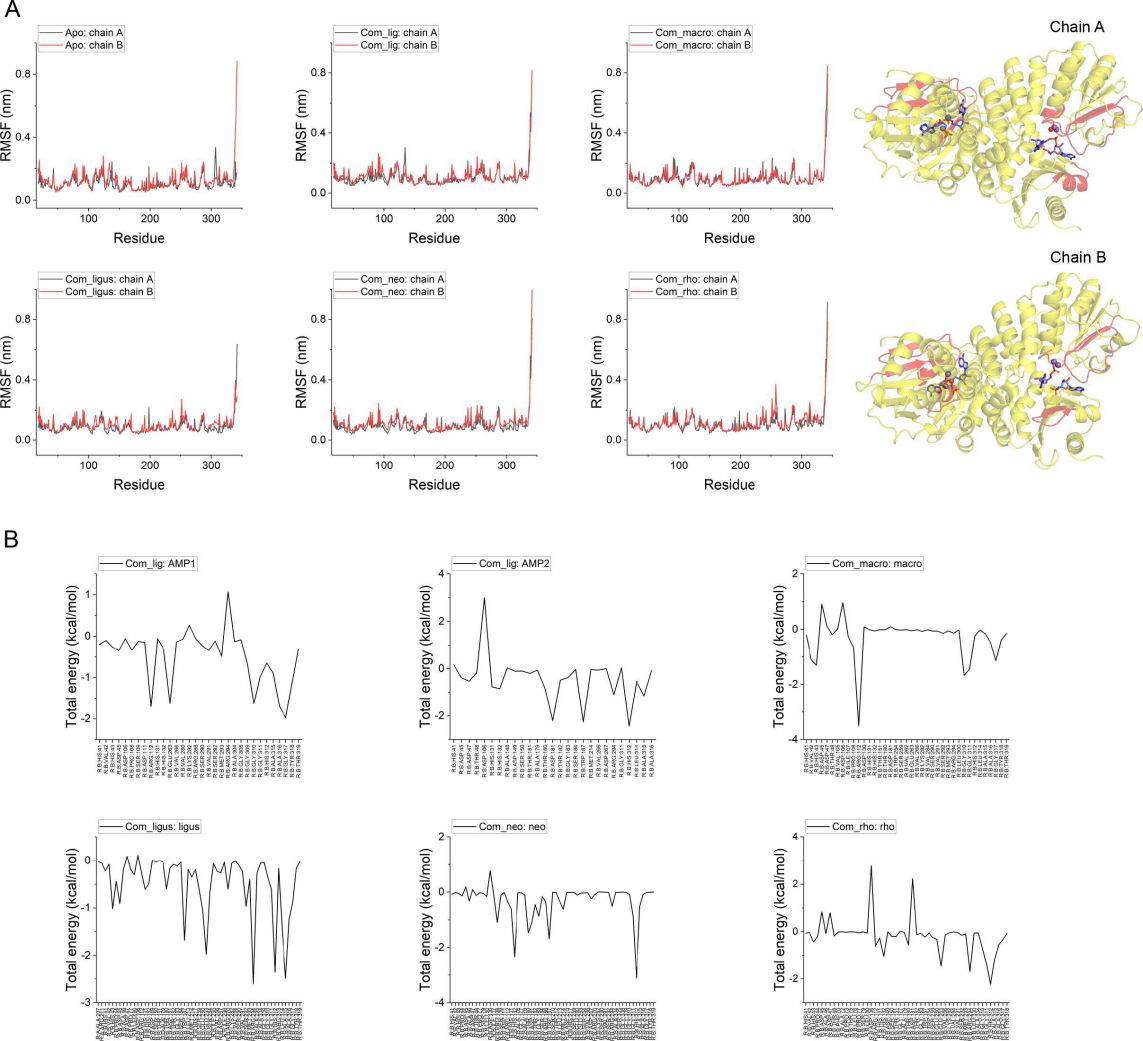
Conformational restraints imposed by natural product inhibitors on CdnP active sites. (**A**) RMSF analysis of apo-CdnP compared with five ligand-bound complexes. The CdnP structure is displayed as a cartoon representation (right panel), with key regions exhibiting significant conformational changes highlighted in red: residues 40–50, 125–150, and 300–310 in chain A, and residues 125–150 and 290–300 in chain B. (**B**) Residue-specific energy decomposition analysis of binding interactions between ligands and CdnP.

Further analysis of root mean square fluctuation (RMSF) revealed distinct conformational behaviors across the enzyme structure. Chain A, which accommodates the substrate pApA, exhibited greater conformational flexibility than chain B (which houses the product AMP) in both the apo state and endogenous ligand-bound state. This enhanced flexibility was particularly pronounced in residues 125–150 and 300–310 surrounding the active pocket, likely facilitating accommodation of the sterically demanding substrate relative to the product. Upon compound binding, both chains demonstrated reduced fluctuation, most notably in residues 40–50, 125–150, and 300–310 of chain A, and residues 125–150 and 290–300 of chain B—all positioned proximal to the active site (Fig. 5A). The diminished RMSF values in chain B indicate stable interactions between the inhibitory compounds and CdnP. Concurrently, the restricted mobility of residues in chain A may impose conformational constraints on apo-CdnP during substrate accommodation, potentially affecting catalytic efficiency or product release. In contrast, the endogenous ligands (pApA and AMPs) exerted minimal influence on the flexibility of residues 125–150 in chain A and 290–300 in chain B, thereby preserving the conformational dynamics necessary for catalytic activity and product dissociation.

We further examined conformational changes within the two active pockets during MD simulations by measuring distances between key residue pairs: Asp181-Arg294, Asp181-His312, Ser292-Asp47, and Ser292-His41. In chain A, the active pocket demonstrated significant conformational fluctuations in both the apo state and endogenous ligand-bound state. However, upon compound binding, this pocket exhibited contraction and enhanced structural stability. In chain B, all ligands (both endogenous ligands and inhibitory compounds) reduced conformational fluctuations (for neodiosmin, this stabilizing effect emerged during the latter portion of the simulation after RMSD stabilization) (Fig. S3B).

To assess global structural impacts, we calculated the radius of gyration (Rg) for all systems and its decomposition along three principal axes (RgX, RgY, and RgZ) (Fig.S3C). The X- and Y-axes were defined as hypothetical lines connecting the N-terminus of one chain to the C-terminus of the other, while the Z-axis corresponded to the symmetry axis between the two monomers. Overall Rg values showed no significant differences across all systems, with similar consistency observed for RgZ measurements. Variations in RgX and RgY exhibited near-symmetric patterns, reflecting the structural symmetry between the two chains. Both the apo state and endogenous ligand-bound state demonstrated greater fluctuations in RgX and RgY compared to compound-bound states, consistent with our observations of RMSF patterns and ligand-induced constraints on active pocket mobility. Notably, macrosporusone A exerted comparatively modest effects on these structural parameters.

Additionally, we conducted gmx_MMPBSA calculations to quantify the interaction energetics between CdnP monomers and between CdnP and its ligands (pApA or inhibitory compounds). Results demonstrated that compound binding minimally affected inter-chain affinity (Table S2) while maintaining or enhancing CdnP-pApA interactions (Table S3), potentially due to constrained pocket dynamics. All compounds exhibited stable binding energies, with ligustroflavone and neodiosmin demonstrating particularly favorable interactions (−61.46 ± 6.31 kcal/mol and −46.63 ± 7.56 kcal/mol, respectively) that were comparable to or exceeded those of the endogenous ligand AMP (Table S4). Energy decomposition analysis revealed distinct residue-specific interaction patterns among the compounds, with notable differences even among the three structurally similar flavonoid glucosides (Fig. 5B). These findings suggest that further investigation of structure-activity relationships among flavonoid glucoside analogs would enhance our understanding of their molecular interactions with CdnP.

### Broad-spectrum antibacterial potential of identified natural product inhibitors

**Fig 6:**
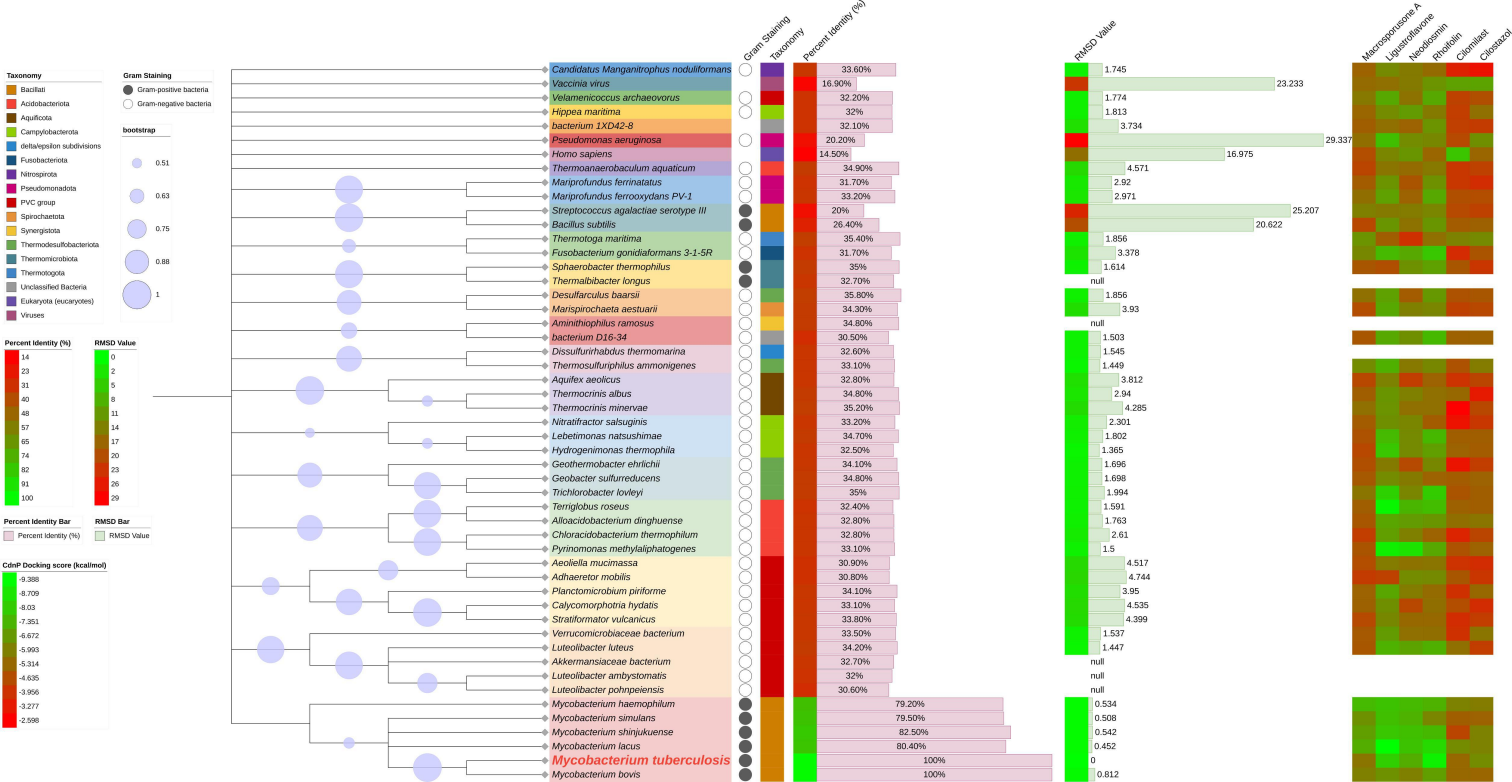
Broad-spectrum antibacterial potential of natural product CdnP inhibitors. The figure presents a comprehensive analysis of CdnP orthologs and inhibitor activity. The left panel contains figure legends, while the right panel displays multiple analyses: a phylogenetic tree of CdnP orthologs with bootstrap values, bacterial taxonomic classification (gram-positive/negative status), sequence identity metrics (represented as both heatmap and bar chart), structural alignment RMSD values (presented as heatmap and bar chart), and molecular docking scores between CdnP orthologs and the identified inhibitory compounds (heatmap).

To explore the potential broad-spectrum antibacterial activity of these compounds, we conducted protein-protein BLAST analysis of *M. tuberculosis* (MTB) CdnP using the UniProt database (with parameters set for 1,000 hits). This analysis revealed homologs across 15 bacterial phyla, including both gram-positive and gram-negative bacteria, with sequence identity ≥29.8% and E-value ≤1e − 33 (Fig. 6; Table S5). Representative sequences from each phylum were selected to construct a phylogenetic tree, enabling analysis of evolutionary relationships between these orthologs and MTB CdnP. The analysis also included previously characterized cyclic dinucleotide phosphodiesterases: three bacterial enzymes (YybT, RocR, and Group B *Streptococcus* (GBS)-CdnP), one mammalian phosphodiesterase (ENPP1), and one viral enzyme (poxin) (25).

As anticipated, orthologs from the *Mycobacterium* genus exhibited high sequence identity with MTB CdnP (all exceeding 79%), while orthologs from other lineages demonstrated considerably lower sequence similarity (below 40%). The human and *Vaccinia virus* orthologs displayed the lowest sequence identity, followed by the other three characterized phosphodiesterases (YybT from *Bacillus subtilis*, RocR from *Pseudomonas aeruginosa*, and GBS-CdnP from *Streptococcus agalactiae*) (Fig. 6; Table S5).

Beyond sequence identity, we sought to assess the three-dimensional structural conservation between CdnP and identified homologs. We obtained structures from the Protein Data Bank or the AlphaFold Database when experimentally determined structures were unavailable. Unfortunately, several homologs lacked both experimental and predicted structures, including orthologs from *Aminithiophilus ramosus*, *Akkermansiaceae bacterium*, *Thermalbibacter longus*, *Luteolibacter ambystomatis*, and *Luteolibacter pohnpeiensis*. Structural alignment of available models against MTB CdnP (PDB: 5JJU) revealed that *Mycobacterium* orthologs exhibited the lowest RMSD values, while all other homologs identified in the BLAST search (identity ≥29.8%) displayed RMSD values below 5. The five previously characterized phosphodiesterases with lower sequence identity demonstrated the highest RMSD values (Fig. 6; Table S5).

The *Pseudomonas aeruginosa* ortholog RocR exhibited the highest RMSD despite not having the lowest sequence identity. This discrepancy likely reflects RocR’s substrate specificity as a cyclic di-GMP-specific phosphodiesterase with minimal activity toward cyclic AMP and cyclic GMP (26). Similarly, the relatively high RMSD values observed for other characterized phosphodiesterases can be explained by their distinct substrate preferences: poxin (*Vaccinia virus*) and ENPP1 (*Homo sapiens*) are 2′,3′-cGAMP-specific phosphodiesterases with limited activity toward 3´,5´-linked cyclic dinucleotides (including bacterial-derived 3´,3´-cGAMP, c-di-AMP, and c-di-GMP) (3, 27, 28). The structural divergence observed for GBS-CdnP (*Streptococcus agalactiae*) and YybT (*Bacillus subtilis*), both of which hydrolyze c-di-AMP and c-di-GMP (29, 30), may arise from limitations in structure prediction, as both models were generated by AlphaFold rather than experimental determination.

We evaluated the inhibitory activity of four natural products and two reference PDE inhibitors (cilomilast and cilostazol) against the identified CdnP orthologs. Three of the four natural products exhibited broad-spectrum inhibition against bacterial CdnP orthologs, with particularly high potency against those from the *Mycobacterium* genus. Macrosporusone A was the exception, demonstrating limited efficacy against orthologs outside this genus (Fig. 6; Table S5). Ligustroflavone demonstrated exceptional inhibitory activity across bacterial CdnP orthologs while exhibiting minimal activity against the 2′,3′-cGAMP-specific PDEs, poxin (*Vaccinia virus*) and ENPP1 (*Homo sapiens*), suggesting favorable selectivity and potentially reduced toxicity. Additionally, ligustroflavone effectively inhibited the cyclic di-GMP-specific phosphodiesterase RocR from *Pseudomonas aeruginosa*. In contrast, the reference inhibitors cilomilast and cilostazol demonstrated poor inhibition of bacterial PDEs while exhibiting selective inhibition of 2′,3′-cGAMP-specific PDEs, particularly cilomilast. This selectivity profile may be attributed to cilomilast’s known activity as a PDE4 inhibitor that targets cAMP hydrolysis (31), and ENPP1’s documented, albeit less efficient, catalytic activity toward cAMP (27).

We employed the ADMETlab platform to evaluate the pharmacokinetic and toxicological properties of the identified inhibitors (32). Analysis revealed that all natural products demonstrated suboptimal absorption characteristics. The flavonoid glucosides exhibited favorable distribution, excretion, and toxicity profiles but demonstrated poor metabolic properties. Conversely, the coumarin derivative macrosporusone A showed advantageous metabolism and excretion characteristics but unfavorable distribution and toxicity profiles (Table S6). These findings highlight the necessity for structural modification and optimization of these compounds to enhance their drug-like properties.

## DISCUSSION

TB remained the most lethal infectious disease globally prior to COVID-19 and continues to pose a significant public health threat, yet treatment options remain limited. The standard 6-month regimen—comprising an initial 2-month intensive phase of rifampicin, isoniazid, ethambutol, and pyrazinamide, followed by a 4-month continuation phase of rifampicin and isoniazid—is protracted, expensive, and frequently compromised by poor adherence. The Bacillus Calmette-Guérin vaccine, though the only licensed and globally administered immunization against TB, demonstrates efficacy in young children but limited protection in adolescents and adults (33). Both these therapeutic and preventive approaches were developed approximately half a century and nearly a century ago, respectively (34, 35). The intervening decades have witnessed the emergence of various drug-resistant TB strains, including isoniazid-monoresistant TB, rifampicin-resistant TB, MDR-TB, pre-extensively drug-resistant TB, and extensively drug-resistant TB, further constraining treatment options (36).

Beyond conventional antibiotic development targeting *M. tuberculosis*, alternative therapeutic strategies have emerged, including host-directed therapy (HDT), which aims to enhance host immune responses or modulate excessive inflammation. Despite its promise, HDT presents considerable challenges, including optimal intervention timing, management of immune risk factors, and differentiation among diverse TB endotypes characterized by distinct immune, epigenetic, metabolic, molecular, and transcriptional signatures (35).

CdnP (Rv2837c) has emerged as a promising anti-TB target due to its role in bacterial virulence and modulation of host STING-dependent interferon responses. Following infection, *M. tuberculosis* releases bacterial CDNs, specifically c-di-AMP, into the host cytosol. Concurrently, the cytosolic DNA sensor cGAMP synthase generates host 2′,3′-cGAMP. These CDNs collectively trigger type I IFN responses through STING-dependent pathways. CdnP counteracts this host defense mechanism by degrading both bacterial-derived c-di-AMP and host-derived 2′,3′-cGAMP, thereby inhibiting STING–interferon regulatory factor 3 (IRF3) activation and IFN secretion. Notably, CdnP mutation significantly reduces *M. tuberculosis* virulence and pathogenicity (analogous to the effect of host PDE ENPP1 deficiency) without compromising bacterial growth *in vitro* (3).

Given CdnP’s host-directed mechanism of action, inhibitors targeting this enzyme represent a promising therapeutic strategy, either as an alternative or adjunctive treatment for drug-resistant TB. These inhibitors could potentiate host cytosolic surveillance pathways while bypassing conventional antibiotic resistance mechanisms that typically affect bactericidal targets (37, 38). Moreover, since CdnP inhibitors impose minimal selective pressure on *M. tuberculosis* survival, they may reduce the likelihood of drug resistance development. Combined administration with direct-acting antimycobacterial agents could potentially abbreviate treatment duration and enhance patient adherence, thereby addressing another major source of drug-resistant *M. tuberculosis* strains.

To date, only three studies have explored CdnP inhibitors, all conducted by the research groups of Herman O. Sintim and William R. Bishai. Their initial investigation elucidated the immune evasion mechanism of CdnP and developed several nonhydrolyzable dinucleotide mimetics as potential inhibitors. Among six analogs tested, Ap(S)A demonstrated competitive inhibition against CdnP while also exhibiting modest activity against ENPP1. Additionally, they established that FDA-approved PDE inhibitors—including cilostazol, cilomilast, sildenafil, and tadalafil (used as reference compounds in our study)—inhibit CdnP. Despite characterizing Ap(S)A’s *in vitro* activity, this study lacked *in vivo* efficacy data (3). Their subsequent publication focused on a high-throughput screening assay (as employed in our study) that identified six CdnP inhibitors, with compound C82 demonstrating the highest potency (IC_50_ 17.5 ± 1.4 µM) (25). Most recently, they conducted virtual screening against the NCI compound library to identify CdnP and ENPP1 inhibitors with favorable oral bioavailability. These compounds were proposed as potential STING pathway potentiators for host-directed tuberculosis therapy, with NCI 79195 exhibiting the highest potency against CdnP (IC_50_ 9.66 µM) (39). While these studies have established CdnP’s druggability and demonstrated preliminary efficacy of CdnP inhibitors against *M. tuberculosis*, they lack robust *in vivo* evidence. Further research is urgently needed to expand the repertoire of CdnP inhibitor scaffolds, elucidate their mechanisms of action, and conduct comprehensive *in vitro* and *in vivo* studies.

In this study, we identified four natural CdnP inhibitors from our in-house compound library and commercial collections: one coumarin derivative (macrosporusone A) and three flavonoid glucosides (ligustroflavone, neodiosmin, and rhoifolin). These compounds demonstrated significant inhibitory activity and binding affinity for CdnP in enzymatic and SPR assays. Molecular dynamics simulations revealed that these inhibitors preferentially and competitively bind to the product (AMP)-binding active site while simultaneously restricting conformational expansion of the substrate (pApA)-binding site. In contrast, CdnP complexed with endogenous ligands (AMP and pApA) maintained considerable conformational flexibility, as evidenced by RMSF analysis, active site dimensional measurements, and radius of gyration parameters (RgX and RgY). Optimal enzyme function typically requires a degree of conformational flexibility, particularly for substrate accommodation and product release, as observed in endogenous ligand-bound CdnP. The identified inhibitors constrain the substrate-binding active site and impede its expansion, thereby disrupting CdnP function. This dual mechanism—competitive inhibition of the product-binding site coupled with restricted flexibility of the substrate-binding site—likely enhances inhibitory efficacy against CdnP. Given CdnP’s role in immune evasion, these compounds may potentiate STING-mediated interferon responses and attenuate *M. tuberculosis* virulence, similar to effects observed in CdnP-deficient bacterial strains (3). These natural products expand the structural diversity of CdnP inhibitor scaffolds, while our mechanistic insights provide valuable direction for future structural optimization efforts.

Our analysis revealed an intriguing structural phenomenon: endogenous ligand-bound CdnP functions as an asymmetric dimer, evidenced by differential RMSD patterns of the four catalytic Mn²^+^ ions, whereas apo-CdnP maintains symmetric positioning of these ions. This observation raises a fundamental question regarding whether the asymmetry in ligand-bound CdnP results from asymmetric ligand-binding events or whether CdnP inherently functions as an asymmetric dimer. In other words, it remains unclear whether CdnP can exist in symmetric ligand-bound states (e.g., with both chains in either pApA-bound or AMP-bound configurations). Previous studies have established that EAL (Glu-Ala-Leu)-type c-di-GMP-specific PDEs also coordinate Mg²^+^ or Mn²^+^ ions within their active sites, with metal ion positioning directly coupled to catalytic activity (40). Interestingly, ligustroflavone binding enhanced the asymmetry of the Mn²^+^ ions, decreasing their RMSD in compound-binding chain B while increasing it in substrate-binding chain A, relative to endogenous ligand-bound CdnP. The potential correlation between this altered metal ion disposition and ligustroflavone’s inhibitory mechanism warrants further investigation.

Evolutionary analysis of CdnP orthologs revealed that our identified natural products exhibit broad antibacterial activity while demonstrating minimal inhibition of host-derived 2′,3′-cGAMP-specific phosphodiesterases. Among the four compounds, ligustroflavone demonstrated superior performance, followed by rhoifolin and neodiosmin, suggesting enhanced capability of flavonoid glucosides in CdnP ortholog inhibition. The coumarin derivative macrosporusone A exhibited potent inhibitory activity exclusively against *Mycobacterium* genus orthologs, potentially positioning it as a candidate for narrow-spectrum or species-specific antimicrobial development. Despite their structural similarities, the three flavonoid glucosides displayed diverse binding modes with CdnP, as demonstrated by molecular docking (Fig. 4) and molecular dynamics simulations (Fig. 6B), indicating the need for comprehensive structure-activity relationship studies. In contrast, FDA-approved PDE inhibitors cilomilast and cilostazol specifically inhibited host-derived 2′,3′-cGAMP-specific phosphodiesterases while exhibiting minimal activity against bacterial orthologs. This differential selectivity suggests that current FDA-approved PDE inhibitors may not be suitable as antibacterial agents and further validates the potential of our identified natural products for antimicrobial therapy development.

Natural products have consistently served as valuable resources for clinical drug development across diverse therapeutic areas, particularly in oncology and infectious disease (7, 41). Coumarins and their derivatives exhibit a broad spectrum of biological activities, including anticancer, antimicrobial, and cytoprotective properties. Similarly, flavonoids demonstrate significant anti-inflammatory, anticarcinogenic, and cardiovascular protective effects, largely attributed to their potent antioxidant capabilities. The glycoside moiety present in flavonoid structures is thought to enhance their pharmacological activity through improved aqueous solubility (42–47). While natural products often exhibit pleiotropic biological activities, further investigation is necessary to confirm their specific inhibitory effects on CdnP.

In summary, this study has identified and characterized four natural product inhibitors of CdnP (Rv2837c) from *M. tuberculosis*: the coumarin derivative macrosporusone A and three flavonoid glucosides (ligustroflavone, neodiosmin, and rhoifolin). These compounds demonstrated significant inhibitory activity against CdnP in enzymatic and SPR assays, with ligustroflavone exhibiting superior performance. Molecular dynamics simulations revealed their dual mechanism of action: competitive binding to the product (AMP)-binding site while simultaneously restricting conformational flexibility of the substrate (pApA)-binding domain. Evolutionary analysis demonstrated their broad-spectrum activity against bacterial CdnP orthologs, particularly those from the *Mycobacterium* genus, while exhibiting minimal inhibition of host phosphodiesterases, suggesting favorable selectivity and potentially reduced toxicity. Unlike FDA-approved PDE inhibitors, which showed poor performance against bacterial orthologs, these natural products represent promising scaffolds for developing novel anti-tuberculosis therapeutics that could enhance host STING-mediated immune responses without exerting selective pressure that promotes antimicrobial resistance. Future studies should focus on structural optimization of these compounds and evaluation of their efficacy in preclinical tuberculosis models to advance their development as potential host-directed therapies for drug-resistant tuberculosis.

## MATERIALS AND METHODS

### Virtual screening and molecular docking

Virtual screening and molecular docking analyses were performed using Schrödinger Maestro software suite (Schrödinger Release 2023-1: Maestro, Schrödinger, LLC, New York, NY, 2023). Protein structures were obtained from the PDB and subsequently optimized using the Protein Preparation Workflow module to ensure structural integrity and appropriate protonation states. Compound libraries underwent preprocessing via the LigPrep module to generate energetically minimized 3D conformations with appropriate stereochemistry. The receptor grid was generated with its center positioned at the active site of the target protein. Initial compound filtering was conducted using high-throughput virtual screening, followed by more rigorous standard precision molecular docking, both implemented with default parameters. Protein-ligand interactions and structural alignments were analyzed and visualized using PyMOL software.

### Protein production and purification

The gene encoding CdnP from *M. tuberculosis* (UniProt accession: P71615) was commercially synthesized (GENEWIZ, Azenta Life Sciences) and subsequently cloned into the pET32a expression vector using NcoI and HindIII restriction sites.

For protein expression, *Escherichia coli* BL21(DE3) cells (Weidibio, cat # EC1002) were transformed with the recombinant plasmid and cultured in Luria-Bertani medium containing appropriate antibiotics at 37°C until reaching an optical density (OD_600_) of 0.6–0.8. Protein expression was induced with 0.4 mM isopropyl β-D-1-thiogalactopyranoside, followed by incubation at 18°C for 16–20 hours. Bacterial cells were harvested by centrifugation, and the resulting pellets were resuspended in buffer A (20 mM Tris-HCl, pH 8.0, 150 mM NaCl, 10 mM imidazole, and 5 mM β-mercaptoethanol).

Cells were lysed by sonication, and cellular debris was removed by ultracentrifugation (10,000 *g*, 4°C, 1 hour). The clarified supernatant was applied to a Ni-NTA affinity column (Qiagen), washed with 20 mL of buffer A, and the recombinant protein was eluted with 15 mL of buffer B (20 mM Tris-HCl, pH 8.0, 150 mM NaCl, 300 mM imidazole, and 5 mM β-mercaptoethanol). The eluted protein was further purified by size-exclusion chromatography (Superdex 75 Increase 10/300 Gl, Cytiva) and stored in a buffer containing 20 mM Tris-HCl, pH 8.0, 150 mM NaCl, 5 mM β-mercaptoethanol, and 20% (vol/vol) glycerol for subsequent experiments.

### High-throughput enzymatic activity assay

The high-throughput screening protocol involved pre-incubation of CdnP (100 nM, final concentration) with test compounds (500 µM, final concentration) or DMSO (dimethyl sulfoxide, negative control) in a buffer containing 50 mM Tris (pH 8.0) and 5 mM MnCl_2_ for 30 minutes at room temperature. The enzymatic reaction was initiated by transferring 44.5 µL of the pre-incubated mixture to each well of a black 96-well plate containing 5.5 µL of reaction solution (10 mM KI, 100 µM coralyne, and 15 µM c-di-AMP, final concentrations). Enzyme activity was monitored through fluorescence measurements (λ_ex_ = 420 nm, λ_em_ = 475 nm) over 30 minutes at 37°C, with reaction rates correlating directly to residual CdnP activity. The complete reaction mixture contained 50 mM Tris (pH 8.0), 5 mM MnCl_2_, 100 nM CdnP, 10 mM KI, 100 µM coralyne, 15 µM c-di-AMP, and 500 µM test compound. IC_50_ values were determined under identical conditions using serial dilutions of test compounds, with DMSO as the negative control.

### SPR assay

Binding affinity between CdnP and test compounds was determined using a Biacore 1K instrument (Cytiva). Recombinant CdnP was immobilized on a Series S CM5 sensor chip according to manufacturer’s protocols. Association and dissociation kinetics were measured using a pre-established multi-cycle kinetics/affinity protocol with eight serially diluted compound concentrations in running buffer (10 mM HEPES, pH 7.4, 150 mM NaCl, 3 mM EDTA, 0.005% [vol/vol] Surfactant P20, and 5% [vol/vol] DMSO). Binding data were analyzed using the multi-cycle kinetics model to determine association rate (K_a_), dissociation rate (K_d_), and equilibrium dissociation constant (K_D_) values.

### MD simulation

MD simulations were performed using CUDA-accelerated GROMACS-2022.2 software with the amber14sb_OL15_corrected-Na-cation-params force field for protein components and the GAFF force field for ligands. Ligand topology files were generated using Sobtop and Multiwfn software packages (48, 49). Initial coordinates for MD simulations were derived from protein-ligand complex structures obtained from molecular docking results. Each complex was solvated in a cubic periodic box using the TIP3P water model, and system charges were neutralized by adding sodium and chloride ions to achieve a physiological ionic strength of 0.15 M. Energy minimization was performed using the conjugate gradient method for 1,000 steps to eliminate unfavorable contacts. System equilibration was conducted sequentially in canonical isothermal-isobaric (NPT) ensembles at 298.15 K and 1 bar, respectively. Production MD simulations were executed for 100 ns on the equilibrated systems. Trajectory analyses were performed using GROMACS built-in modules, with subsequent data processing and visualization accomplished using Origin 2021, PyMOL, and VMD software.

Free energy calculations and energy decomposition analyses were conducted using the gmx_MMPBSA program (50). Frames were extracted at 20 ps intervals beginning from system equilibration, with all other parameters maintained at default settings.

### BLAST and evolutionary analyses

Using the *M. tuberculosis* CdnP (Rv2837c) sequence as a query, we performed a protein-protein BLAST search against the UniProt database with a limit of 1,000 hits and default parameters. From these results, representative sequences from each bacterial phylum were selected and combined with five previously characterized phosphodiesterases (Yybt, RocR, GBS-CdnP, ENPP1, and poxin) for comparative analysis. Multiple sequence alignment of 51 CdnP orthologs was conducted using the ClustalW algorithm implemented in MEGA software (51). Phylogenetic analysis was performed using the neighbor-joining method with the Poisson substitution model and 1,000 bootstrap replicates to ensure statistical robustness. All protein accession numbers are cataloged in Table S5. The resulting phylogenetic tree was visualized, annotated, and formatted using the Interactive Tree Of Life web-based tool (52).

## SUPPLEMENTAL MATERIAL

Supplemental material spectrum.03258-24-s0001.docx Tables S1 to S4; Fig. S1 to S3.

Table S5 spectrum.03258-24-s0002.xlsx Sequence and structural data of CdnP orthologs utilized in BLAST and evolutionary analyses, along with their docking outcomes involving compounds.

Table S6 spectrum.03258-24-s0003.xlsx Assessment of absorption, distribution, metabolism, excretion, and toxicity (ADMET) properties of compounds.

ASM does not own the copyrights to Supplemental Material that may be linked to, or accessed through, an article. The authors have granted ASM a non-exclusive, world-wide license to publish the Supplemental Material files. Please contact the corresponding author directly for reuse.
