# Supplementary material for: Discovery of natural CdnP inhibitors through structure-based virtual screening and molecular dynamics simulations
Source: Microbiol Spectr. 2025 Apr 30;13(6):e03258-24. doi: 10.1128/spectrum.03258-24 (PMC12131788; doi:10.1128/spectrum.03258-24)
Supplement: Supplemental material — Tables S1 to S4; Fig. S1 to S3. [file spectrum.03258-24-s0001.docx]

Supporting Information for

**Article**

**Discovery of natural CdnP inhibitors through structure-based virtual screening and molecular dynamics simulations**

Xiaoxia Gu^†, 1, 2^, Chaohu Xiong^†, 2^, Xinyu Wang^2^, Hucheng Zhu^2^, Weiguang Sun*^, 2^, Yan He*^, 1^, Jinwen Zhang*^, 1^

^1^Department of Pharmacy, Tongji Hospital, Tongji Medical College, Huazhong University of Science and Technology, Wuhan, People’s Republic of China

^2^Hubei Key Laboratory of Natural Medicinal Chemistry and Resource Evaluation, School of Pharmacy, Tongji Medical College, Huazhong University of Science and Technology, Wuhan, People’s Republic of China

^†^These authors contributed equally to the work.

*Correspondence: weiguang_sun@hust.edu.cn (Weiguang Sun), [heyan_may@hotmail.com (Yan He),](mailto:heyan_may@hotmail.com,) tjzhangjinwen@163.com (Jinwen Zhang).

**
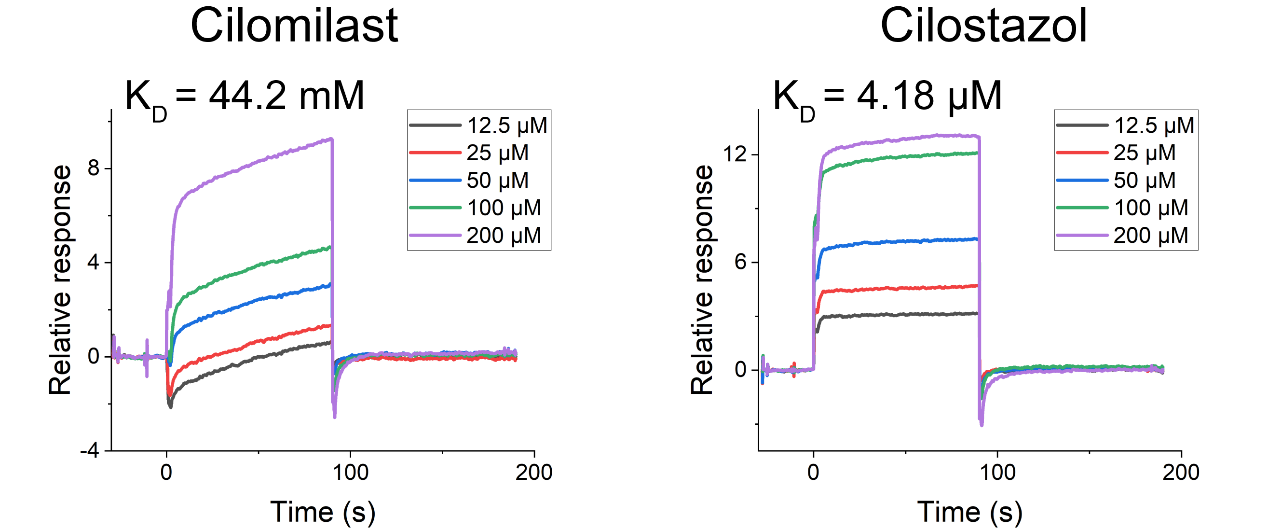
**

**FIG S1** Binding affinity analysis between FDA-approved PDE inhibitors (cilomilast and cilostazol) and mycobacterial CdnP.

**
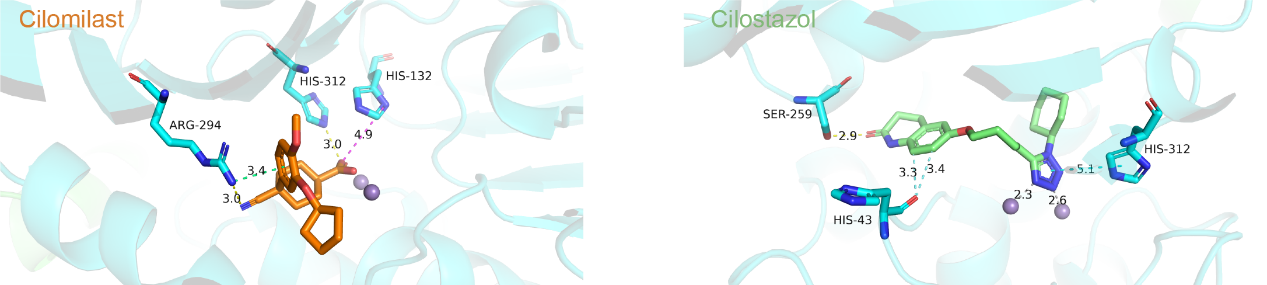
**

**FIG S2** Molecular docking analysis of FDA-approved PDE inhibitors (cilomilast and cilostazol) with mycobacterial CdnP.

**
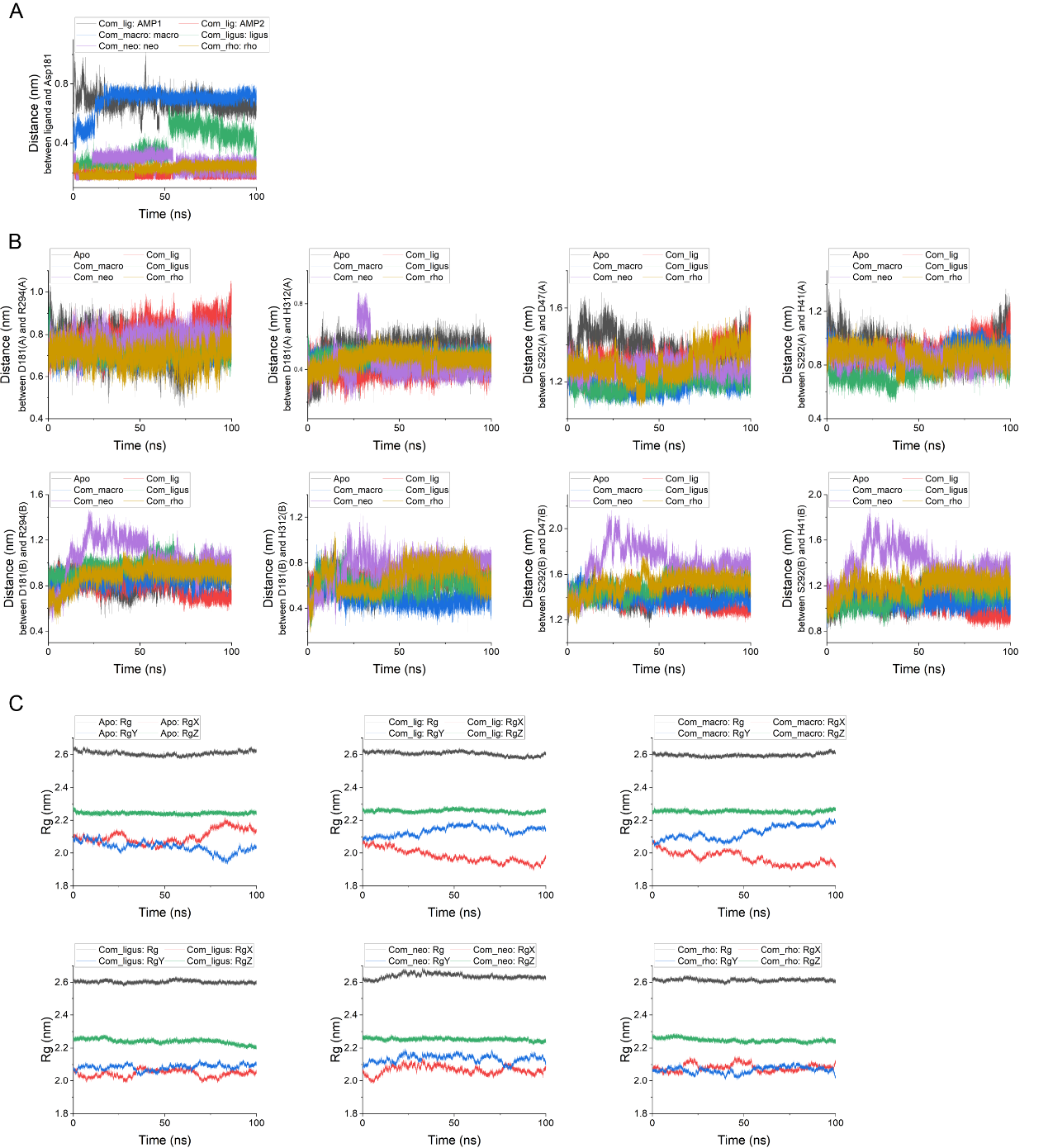
**

**FIG S3** Molecular dynamics simulation analysis of apo CdnP and ligand-bound complexes. (A) Distance measurements between ligands from five complex groups and Asp181 in chain B. (B) Binding pocket size analysis in both chains, determined by measuring distances between key residue pairs: Asp181-Arg294, Asp181-His312, Ser292-Asp47, and Ser292-His41. (C) Radius of gyration (Rg) plots comparing apo CdnP and the five ligand-bound complex groups.

**TABLE S1** Surface plasmon resonance (SPR) binding kinetics and affinity parameters for interactions between CdnP and identified inhibitory compounds.

| **Compound** | **Quality kinetics Chi^2^ (RU^2^)** | **1:1 binding k_a_ (1/Ms)** | **k_d_ (1/s)** | **K_D_ (M)** | **Rmax (RU)** | **tc** |
| --- | --- | --- | --- | --- | --- | --- |
| Macrosporusone A | 2.46e+00 | 2.54e+05 | 1.62e-07 | 6.37e-13 | -3.3 | 1.02e+08 |
| Ligustroflavone | 4.90e-02 | 3.39e+02 | 6.42e-06 | 1.89e-08 | 1.0 | 1.16e+08 |
| Neodiosmin | 8.92e-02 | 5.95e+00 | 2.29e-03 | 3.84e-04 | 26.8 | 1.17e+08 |
| Rhoifolin | 2.02e-01 | 2.15e+05 | 2.09e-01 | 9.72e-07 | -2.1 | 1.49e+08 |
| Cilomilast | 3.17e-01 | 1.05e+01 | 4.65e-01 | 4.42e-02 | 1733.5 | 6.30e+06 |
| Cilostazol | 7.64e-02 | 7.67e+04 | 3.21e-01 | 4.18e-06 | -1.9 | 5.48e+09 |

**TABLE S2** Binding affinity analysis between chains A and B with energy decomposition for apo CdnP and five ligand-bound complex groups.

| **Delta (Complex - Receptor - Ligand): chain A-chain B** | **Apo** | **Com_lig** | **Com_macro** | **Com_ligus** | **Com_neo** | **Com_rho** |
| --- | --- | --- | --- | --- | --- | --- |
| Energy Component | Average ± SD (kcal/mol) | | | | | |
| ΔBOND | -0.00 ± 0.00 | -0.00 ± 0.00 | 0.00 ± 0.00 | -0.00 ± 0.00 | 0.00 ± 0.00 | 0.00 ± 0.00 |
| ΔANGLE | 0.00 ± 0.00 | 0.00 ± 0.00 | -0.00 ± 0.00 | -0.00 ± 0.00 | 0.00 ± 0.00 | 0.00 ± 0.00 |
| ΔDIHED | -0.00 ± 0.00 | -0.00 ± 0.00 | 0.00 ± 0.00 | 0.00 ± 0.00 | 0.00 ± 0.00 | -0.00 ± 0.00 |
| ΔVDWAALS | -227.27 ± 8.03 | -233.09 ± 8.61 | -228.47 ± 8.42 | -236.48 ± 7.63 | -224.94 ± 7.65 | -230.13 ± 8.60 |
| ΔEEL | -32.59 ± 47.90 | -61.66 ± 41.83 | -83.70 ± 29.51 | -64.10 ± 31.85 | 0.25 ± 44.25 | -60.69 ± 37.67 |
| Δ1-4 VDW | -0.00 ± 0.00 | 0.00 ± 0.00 | -0.00 ± 0.00 | -0.00 ± 0.00 | -0.00 ± 0.00 | -0.00 ± 0.00 |
| Δ1-4 EEL | 0.00 ± 0.00 | -0.00 ± 0.00 | 0.00 ± 0.00 | 0.00 ± 0.00 | 0.00 ± 0.00 | 0.00 ± 0.00 |
| ΔEGB | 90.28 ± 42.66 | 118.19 ± 35.63 | 135.41 ± 26.50 | 119.86 ± 27.87 | 56.94 ± 40.16 | 117.04 ± 34.66 |
| ΔESURF | -30.71 ± 0.88 | -31.47 ± 0.77 | -31.10 ± 0.69 | -31.85 ± 0.59 | -30.34 ± 0.64 | -31.14 ± 0.87 |
|  |  |  |  |  |  |  |
| ΔGGAS | -259.85 ± 48.64 | -294.75 ± 41.24 | -312.17 ± 29.76 | -300.58 ± 31.63 | -224.69 ± 43.90 | -290.82 ± 38.81 |
| ΔGSOLV | 59.57 ± 42.23 | 86.72 ± 35.42 | 104.31 ± 26.26 | 88.01 ± 27.67 | 26.59 ± 39.87 | 85.90 ± 34.20 |
|  |  |  |  |  |  |  |
| ΔTOTAL | -200.28 ± 11.10 | -208.03 ± 11.16 | -207.86 ± 8.77 | -212.57 ± 9.55 | -198.10 ± 9.27 | -204.92 ± 9.51 |

**TABLE S3** Binding affinity and energy decomposition analysis between CdnP and pApA across five ligand-bound complex groups.

| **Delta (Complex - Receptor - Ligand): Protein_pApA** | **Com_lig** | **Com_macro** | **Com_ligus** | **Com_neo** | **Com_rho** |
| --- | --- | --- | --- | --- | --- |
| Energy Component | Average ± SD (kcal/mol) | | | | |
| ΔBOND | 0.00 ± 0.00 | -0.00 ± 0.00 | 0.00 ± 0.00 | 0.00 ± 0.00 | 0.00 ± 0.00 |
| ΔANGLE | 0.00 ± 0.00 | 0.00 ± 0.00 | -0.00 ± 0.00 | 0.00 ± 0.00 | -0.00 ± 0.00 |
| ΔDIHED | 0.00 ± 0.00 | -0.00 ± 0.00 | 0.00 ± 0.00 | -0.00 ± 0.00 | -0.00 ± 0.00 |
| ΔVDWAALS | -67.72 ± 4.32 | -75.76 ± 4.65 | -68.12 ± 3.94 | -73.83 ± 3.88 | -71.52 ± 4.34 |
| ΔEEL | 112.65 ± 6.11 | 107.74 ± 4.48 | 114.83 ± 4.90 | 108.02 ± 4.86 | 117.57 ± 8.94 |
| Δ1-4 VDW | 0.00 ± 0.00 | 0.00 ± 0.00 | 0.00 ± 0.00 | -0.00 ± 0.00 | 0.00 ± 0.00 |
| Δ1-4 EEL | 0.00 ± 0.00 | 0.00 ± 0.00 | 0.00 ± 0.00 | -0.00 ± 0.00 | -0.00 ± 0.00 |
| ΔEGB | -113.21 ± 6.86 | -105.52 ± 4.42 | -113.12 ± 5.34 | -109.11 ± 4.93 | -119.20 ± 11.09 |
| ΔESURF | -7.81 ± 0.32 | -8.16 ± 0.20 | -7.82 ± 0.20 | -8.17 ± 0.23 | -7.96 ± 0.36 |
|  |  |  |  |  |  |
| ΔGGAS | 44.92 ± 7.57 | 31.98 ± 5.52 | 46.71 ± 5.99 | 34.19 ± 5.66 | 46.05 ± 10.92 |
| ΔGSOLV | -121.02 ± 6.85 | -113.67 ± 4.43 | -120.94 ± 5.37 | -117.28 ± 4.84 | -127.16 ± 10.88 |
|  |  |  |  |  |  |
| ΔTOTAL | -76.10 ± 4.01 | -81.69 ± 4.18 | -74.23 ± 3.94 | -83.09 ± 3.53 | -81.11 ± 4.33 |

**TABLE S4** Binding affinity and energy decomposition analysis between CdnP and inhibitory ligands across five distinct complex groups.

| **Delta (Complex - Receptor - Ligand): Protein_ligand** | **Com_lig**  **(AMP1)** | **Com_lig**  **(AMP2)** | **Com_macro** | **Com_ligus** | **Com_neo** | **Com_rho** |
| --- | --- | --- | --- | --- | --- | --- |
| Energy Component | Average ± SD (kcal/mol) | | | | | |
| ΔBOND | 0.00 ± 0.00 | 0.00 ± 0.00 | -0.00 ± 0.00 | -0.00 ± 0.00 | -0.00 ± 0.00 | -0.00 ± 0.00 |
| ΔANGLE | -0.00 ± 0.00 | 0.00 ± 0.00 | 0.00 ± 0.00 | 0.00 ± 0.00 | 0.00 ± 0.00 | 0.00 ± 0.00 |
| ΔDIHED | 0.00 ± 0.00 | 0.00 ± 0.00 | -0.00 ± 0.00 | 0.00 ± 0.00 | -0.00 ± 0.00 | 0.00 ± 0.00 |
| ΔVDWAALS | -30.65 ± 4.14 | -33.65 ± 3.45 | -35.33 ± 2.78 | -62.31 ± 6.45 | -51.33 ± 7.58 | -42.02 ± 4.43 |
| ΔEEL | 78.74 ± 7.94 | 160.99 ± 3.73 | 64.02 ± 2.36 | -1.62 ± 2.07 | 7.35 ± 3.18 | 0.39 ± 3.07 |
| Δ1-4 VDW | -0.00 ± 0.00 | -0.00 ± 0.00 | 0.00 ± 0.00 | 0.00 ± 0.00 | -0.00 ± 0.00 | 0.00 ± 0.00 |
| Δ1-4 EEL | 0.00 ± 0.00 | 0.00 ± 0.00 | -0.00 ± 0.00 | -0.00 ± 0.00 | 0.00 ± 0.00 | 0.00 ± 0.00 |
| ΔEGB | -85.57 ± 10.15 | -169.23 ± 4.35 | -54.83 ± 1.77 | 10.15 ± 1.70 | 3.74 ± 3.41 | 16.56 ± 2.63 |
| ΔESURF | -3.69 ± 0.50 | -4.34 ± 0.26 | -4.28 ± 0.18 | -7.67 ± 0.66 | -6.39 ± 0.80 | -6.12 ± 0.44 |
|  |  |  |  |  |  |  |
| ΔGGAS | 48.09 ± 6.81 | 127.33 ± 4.76 | 28.69 ± 3.62 | -63.94 ± 6.60 | -43.98 ± 9.63 | -41.63 ± 4.33 |
| ΔGSOLV | -89.26 ± 10.45 | -173.57 ± 4.42 | -59.11 ± 1.74 | 2.48 ± 1.40 | -2.65 ± 2.84 | 10.44 ± 2.60 |
|  |  |  |  |  |  |  |
| ΔTOTAL | -41.17 ± 7.03 | -46.24 ± 4.20 | -30.42 ± 2.91 | -61.46 ± 6.31 | -46.63 ± 7.56 | -31.19 ± 3.99 |
